# Monitoring emissions from the 2015 Indonesian fires using CO satellite data

**DOI:** 10.1098/rstb.2017.0307

**Published:** 2018-10-08

**Authors:** Narcisa Nechita-Banda, Maarten Krol, Guido R. van der Werf, Johannes W. Kaiser, Sudhanshu Pandey, Vincent Huijnen, Cathy Clerbaux, Pierre Coheur, Merritt N. Deeter, Thomas Röckmann

**Affiliations:** 1 Institute for Marine and Atmospheric Research Utrecht (IMAU), University of Utrecht, 3584 CC Utrecht, The Netherlands; 2 Department of Meteorology and Air Quality (MAQ), Wageningen University and Research Centre, 6700 AA Wageningen, The Netherlands; 3 SRON Netherlands Institute for Space Research, 3584 CA Utrecht, The Netherlands; 4 Faculty of Science, Vrije Universiteit, 1081 HV Amsterdam, The Netherlands; 5 Air Chemistry Department, Max Planck Institute for Chemistry, 55128 Mainz, Germany; 6 Royal Netherlands Meteorological Institute (KNMI), 3731 GA De Bilt, The Netherlands; 7 LATMOS/IPSL, Sorbonne Université, Université Paris-Saclay, CNRS, 75252 Paris, France; 8 Spectroscopie de l'Atmosphère, Service de Chimie Quantique et Photophysique, Université Libre de Bruxelles (ULB), 1050 Brussels, Belgium; 9 National Center for Atmospheric Research (NCAR), Boulder, CO 80305, USA

**Keywords:** biomass burning, emissions, atmosphere, satellite data, peat

## Abstract

Southeast Asia, in particular Indonesia, has periodically struggled with intense fire events. These events convert substantial amounts of carbon stored as peat to atmospheric carbon dioxide (CO_2_) and significantly affect atmospheric composition on a regional to global scale. During the recent 2015 El Niño event, peat fires led to strong enhancements of carbon monoxide (CO), an air pollutant and well-known tracer for biomass burning. These enhancements were clearly observed from space by the Infrared Atmospheric Sounding Interferometer (IASI) and the Measurements of Pollution in the Troposphere (MOPITT) instruments. We use these satellite observations to estimate CO fire emissions within an inverse modelling framework. We find that the derived CO emissions for each sub-region of Indonesia and Papua are substantially different from emission inventories, highlighting uncertainties in bottom-up estimates. CO fire emissions based on either MOPITT or IASI have a similar spatial pattern and evolution in time, and a 10% uncertainty based on a set of sensitivity tests we performed. Thus, CO satellite data have a high potential to complement existing operational fire emission estimates based on satellite observations of fire counts, fire radiative power and burned area, in better constraining fire occurrence and the associated conversion of peat carbon to atmospheric CO_2_. A total carbon release to the atmosphere of 0.35–0.60 Pg C can be estimated based on our results.

This article is part of a discussion meeting issue ‘The impact of the 2015/2016 El Niño on the terrestrial tropical carbon cycle: patterns, mechanisms and implications'.

## 1. Introduction

Fires in Indonesia are ignited every year by agricultural activities and land clearing practices [1]. Meteorological conditions often associated with the warm phase of the El Niño–Southern Oscillation can, in some cases, cause the evolution of widespread fires, emitting large quantities of carbon dioxide (CO_2_), other greenhouse gases such as methane (CH_4_) and nitrous oxide (N_2_O), carbon monoxide (CO), nitrogen oxides (NO_X_), volatile organic compounds and aerosols, causing poor air quality in Indonesia and the surrounding region [2,3]. Such an event occurred in 2015, when fires spread rapidly due to the drought conditions associated with the strongest El Niño event in the past two decades [4,5].

The tropical drought conditions typically associated with an El Niño event have a twofold effect on the terrestrial carbon cycle: the ecosystem stress changes photosynthesis and respiration, and the risk and severity of fires are increased [6]. Indonesian fires can become particularly severe in such a situation due to the occurrence of peat substrate. Peat stores large quantities of carbon in the form of partially decayed organic matter that can potentially be released to the atmosphere by fires. Indonesian peatlands alone store about 60 Pg C, which is about 65% of the tropical peatland carbon reservoir [7]. Land clearing fires convert forested land for the cultivation of oil palm, timber and pulp, and are often illegal [8–10]. A substantial part of these forests are on peatland [11]. Peat is, in most cases, drained before clearing and cultivation, making it more susceptible to fires. During extreme drought conditions as in an El Niño situation, peat fires can spread in an uncontrolled manner far beyond the intended area, converting the peat carbon pool to atmospheric CO_2_.

Indonesian fires often emit large quantities of CO by incomplete combustion associated with the occurrence of peat. CO is a major air pollutant that affects the oxidation capacity of the atmosphere through its reaction with the hydroxyl radical (OH) and simultaneously acts as a precursor for ozone formation, an important short-lived greenhouse gas. CO is converted to CO_2_ with a lifetime of one to three months in the atmosphere, which makes it very suitable for detecting emissions from widespread fires. According to the Global Fire Emission Database [12], the 2015 Indonesian fires produced the largest CO emissions from biomass burning since 1997–1998, when another strong El Niño triggered the most severe Indonesian fire event to be recorded since 1990 [4].

Emissions from fires to the atmosphere are typically derived using space-based detection of fire counts (FC), fire radiative power (FRP) or burned area (BA). These emission algorithms estimate the spatial distribution of dry matter (DM) consumed during fire events based on space-based observations of BA in combination with biogeochemical modelling (GFED) or FRP observations (Global Fire Assimilation System, GFAS [13]). This DM estimate is subsequently converted to emissions of CO_2_, CO and NO_X_ based on emission factors and biome distribution [14]. Over Indonesia and elsewhere, estimated emissions are affected by substantial errors due to frequent cloud cover inhibiting detection, and limited knowledge of biome distribution and emission factors. Most importantly, the degree to which fires consume peat challenges the emission models as the depth of burning cannot be easily estimated using remote sensing data.

The 2015 event was recorded from space by several satellite instruments currently providing high-quality CO data: IASI (Infrared Atmospheric Sounding Interferometer) on-board Metop, MOPITT (Measurements of Pollution in the Troposphere) on-board the Terra satellite, the Tropospheric Emission Spectrometer (TES) on-board EOS-Aura and the Atmospheric InfraRed Sounder (AIRS) on-board EOS-Aqua. These satellite products provide a large number of CO total columns, which can be used to monitor fire emissions from an atmospheric perspective. Owing to its residence time in the atmosphere, a CO plume can be observed for up to 1 week after it has been emitted. CO emitted over typically cloudy areas, such as Indonesia and Papua, is likely to be observed while it is transported away from the emission location. Therefore, using CO for monitoring fires would be much less sensitive to the occurrence of clouds than other satellite-based products, such as BA and FRP.

Previous inverse modelling estimates of CO emissions from the 2015 Indonesian fires were made by Huijnen *et al.* [15] and Yin *et al.* [16], who estimated emissions of 84 (September and October 2015) and 122 Tg CO (entire year 2015), respectively. Both studies used MOPITT data, which were shown to have a poorer performance at detecting extreme events compared to IASI [17]. Huijnen *et al.* [15] also used measured emission factors during this event to convert their estimates into CO_2_ and CH_4_ emission estimates. Other estimates for CO_2_ emissions for this event using satellite data were made by Heymann *et al.* [18] and Lohberger *et al.* [19].

We focus here on comparing IASI and MOPITT, which both measure CO in the thermal infrared (TIR), and have relatively high spatial resolution and global coverage. By contrast, TES has a sparser spatial coverage, while AIRS has a larger footprint and measurement error [20,21]. MOPITT differs from IASI in terms of measurement technique, vertical sensitivity and retrieval strategy, having an additional measurement band for CO in the near infrared (NIR). Typical differences up to 20% in the retrieved total columns of the two products have been reported [17].

The widespread 2015 Indonesian fires give us the opportunity to compare the estimated CO emissions of inventories with/against satellite products. To realize an atmospheric-based system for monitoring fire emissions, CO emissions need to be tracked in the atmosphere, where they mix with CO sources from industry and atmospheric oxidation of hydrocarbons, and in which CO is oxidized by atmospheric hydroxyl (OH). The TM5-4DVAR inverse modelling system [22–24] is used in this paper to optimize CO emissions from the Indonesian fires by assimilating satellite CO data from MOPITT or IASI.

In the following sections, we evaluate the performance of emission inventories in capturing the spatial and temporal evolution of CO emissions from the 2015 fire event in Indonesia and compare, for the first time, CO emission estimates for a large biomass burning event, optimized using IASI and MOPITT satellite data. We show the consistency between the emissions derived from these two products and advocate the use of these observations in fire monitoring systems such as the Copernicus atmosphere monitoring service (CAMS) towards constraining fire emissions.

## 2. Material and methods

### (a) IASI data

IASI is a sun-synchronous nadir-viewing Fourier Transform Spectrometer based on Michelson interferometry that flies on-board the Metop-A and Metop-B operational platforms, launched in 2006 and 2012, respectively. IASI measures CO at 4.7 µm, having the highest sensitivity in the mid-troposphere. A total of 120 views are collected over a swath of 2200 km, with footprints of 4 × 12 km diameter pixels (at nadir). Metop crosses the equator at 9.30 local time, each morning and evening.

The IASI CO FORLI (Fast Optimal Retrievals on Layers for IASI) retrieval uses a fixed prior profile everywhere and allows a 30–63% error on each retrieved level, with a 5 km vertical correlation scale [17,25]. Cloud filtering is applied when more than 25% cloudiness is detected. In the tropics, profiles are retrieved with typically 2 degrees of freedom of signal (DOFS).

### (b) MOPITT data

MOPITT is a nadir-viewing instrument that uses gas filter correlation radiometry to measure CO at 4.7 µm (TIR) and 2.3 (NIR) µm. The 2.3 µm band provides data only over land during day-time and typically has a higher sensitivity to the lower troposphere. In this paper, we use MOPITT version 7 Level 2 data from both the joint NIR–TIR product, which combines information from both wavelength regions, and the TIR-only product. MOPITT flies on the sun-synchronous orbiting Terra satellite launched in 1999, crossing the equator at approximately 10.30 local time each morning and evening. It has a swath of 22 × 650 km, with 116 cross-track pixels.

The MOPITT retrieval algorithm assumes lognormal statistics for CO variability and uses a variable *a priori* based on a 1-degree monthly climatology from CAM-chem model simulations [26]. A 30% error is allowed on the prior profile, with a vertical correlation length of 100 hPa. Cloud filtering is applied for more than 5% cloud fraction. DOFS values typically range from 1.1 to 2 for the NIR–TIR product and 1 to 1.6 for the TIR-only product [27]. Validation results for MOPITT V7 NIR–TIR and TIR-only total column values (used below) indicate that retrieval biases are generally less than 0.05 × 10^18^ mol cm^−2^ [26].

### (c) TM5-4DVAR

The TM5-4DVAR model is employed in this study to estimate CO emissions from the 2015 fire event over Indonesia. The model consists of the Tracer Transport Model version 5 (TM5 [24]) and the four-dimensional variational optimization shell. TM5-4DVAR was previously used successfully to optimize CO emissions on global and regional scales using MOPITT [24] and IASI data [23].

**Figure 1.**
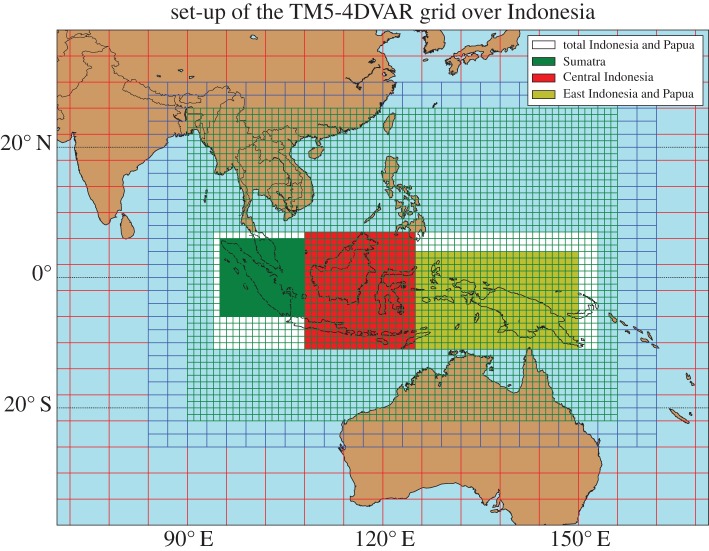
Grid definition in the TM5-4DVAR model over equatorial Asia. The regions analysed in this paper are highlighted in green (Sumatra), red (Central Indonesia including Kalimantan) and yellow (East Indonesia, including Papua).

The TM5 model is driven by offline meteorological fields from the ERA-Interim reanalysis of the European Centre for Medium-range Weather Forecasts (ECMWF) [28]. We run TM5 on a 6° × 4° horizontal resolution and on 25 vertical hybrid sigma-pressure levels. We make use of the TM5 zoom capabilities to increase the horizontal resolution over southeast Asia to 1° × 1°, with an intermediate zoom region of 3° × 2°, as shown in figure 1.

The 4DVAR optimizer finds a set of emissions that minimizes the differences between modelled and observed CO concentrations, as well as the difference with respect to a prior set of emissions, by minimizing the following cost function:
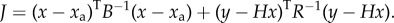
Here, *x* and *x*_a_ are the set of posterior (optimized) and prior emissions. In this study, we optimize for the total grid-scale CO emissions outside the zoom regions on a weekly basis. Inside the zoom regions, the three-daily biomass burning grid-scale CO emissions are optimized, similar to the study by Krol *et al.* [23]. Observations from the global NOAA network of surface stations, as well as satellite data from inside the 3 × 2 zoom region from either MOPITT or IASI, are represented in *y*. Only background stations not influenced by nearby CO emissions are used from the NOAA network, as described in [29]. *H* is the model operator, converting emissions into concentrations sampled at the observation time and location. *B* and *R* are the error covariance matrixes for the prior emissions and the observations, respectively. A relative error of 250% is assumed for the grid-scale prior emissions. For the global prior emissions, a semi-Gaussian error setting with a horizontal correlation length scale of 1000 km and a 9.5-month correlation time-scale is used. For biomass burning emissions over southeast Asia, a 200 km correlation length scale and 3-day correlation time-scale are used. The nonlinear m1qn3 optimizer from Gilbert & Lemaréchal [30] is employed to avoid negative emissions.

When comparing observed and modelled columns, the model is sampled at the observation locations. A model pseudo-profile is then built, taking into account the satellite instruments’ sensitivity to the vertical layers of the atmosphere, described by the averaging kernels (AKs) and the *a priori* retrieval profile:
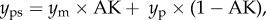
where 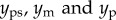 are the model pseudo-profile, the actual model profile and the *a priori* profile of CO mixing ratio in the case of IASI. In the case of MOPITT, the logarithm of CO mixing ratio is used in the above equation instead of the mixing ratio [26]. The pseudo-profile is then integrated over the vertical to obtain a total column, which can be compared to the satellite-observed column.

### (d) Simulation set-up

We use the MACCity anthropogenic emissions and CO oxidation based on OH climatological fields from Spivakovsky *et al.* [31] scaled with a global factor of 0.92, as recommended in [32] based on methyl chloroform observations. CO chemical production from oxidation of methane (CH_4_) and from non-methane volatile organic compounds (NMVOCs) is based on a 2010 simulation of the TM5 full chemistry version [32].

The main results described in this paper are from the ‘IASI’ and ‘MOPITT’ simulations, where IASI and, respectively, MOPITT level 2 data are used together with NOAA surface stations, to optimize CO emissions. The IASI day-time only data are used because the day-time FORLI retrievals are more sensitive to lower altitudes than the night-time data [33]. The observation variance is enhanced by a factor of 50 to account for observation error correlation, as suggested in [29]. For MOPITT, we use both day-time and night-time data from the version 7 NIR–TIR product. No error inflation is typically applied for MOPITT.

A set of tests was performed to evaluate the sensitivity of the resulting emissions with respect to the data used and the model set-up (electronic supplementary material, table S1). In the ‘MOPITT J day’ and ‘MOPITT T day’, only day-time data were used from MOPITT from the NIR–TIR and TIR-only products, respectively. In ‘MOPITT infl’, we tested the impact of applying an error inflation factor of 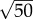 for MOPITT, as done for IASI.

IASI has a wider swath and a better spatial coverage than MOPITT, which can be seen also in the animations in the electronic supplementary material. We tested whether the difference in spatial coverage between IASI and MOPITT could impact the resulting emissions in the ‘IASI filt’ simulation, where we left out IASI data from locations more than 1° latitude or 1° longitude away from MOPITT observations from the same day.

To test the potential magnitude of the effect that changes in OH due to the Indonesian fires could have on estimated emissions [34], we performed two sensitivity tests, ‘IASI OH’ and ‘IASI OH GFED,’ using modified OH fields. In these simulations, standard OH fields were scaled with the daily three-dimensional ratio between two global chemistry simulations with the Composition Integrated Forecasting System (C-IFS) model [15,35], with and without peat emissions of CO, NOx and NMVOC. Most simulations presented use GFAS v.1.3 [13] as prior biomass burning emissions, except for ‘IASI OH GFED’, which uses GFED4s [12] as prior.

## 3. Results

### (a) MOPITT and IASI CO columns

**Figure 2.**
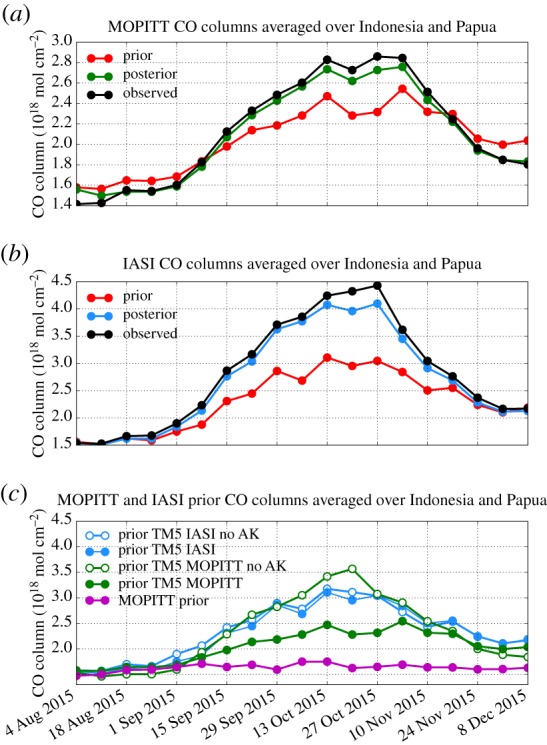
Measured weekly averaged (*a*) MOPITT and (*b*) IASI CO columns over Indonesia and Papua, and modelled by the prior GFAS and posterior IASI and MOPITT inversions. (*c*) Prior TM5 columns for IASI and MOPITT before and after applying the averaging kernel. The MOPITT retrieval prior, originating from a CAM-chem simulation with climatological biomass burning emissions, is also shown.

Figure 2*a*,*b* shows the weekly averaged MOPITT and IASI observed CO columns over Indonesia and Papua (white box in figure 1) and the modelled columns using GFAS prior and optimized emission over the same areas based on each of the two datasets. The IASI CO columns (black line in figure 2*b*) tripled from a background average of 1.5 × 10^18^ mol cm^−2^ in August to about 4.5 × 10^18^ mol cm^−2^ at the end of October, and afterwards decreased again to 2 × 10^18^ mol cm^−2^ in December. Similarly, the MOPITT CO columns (black line in figure 2*a*) doubled from 1.4 × 10^18^ mol cm^−2^ in August to 2.8 × 10^18^ mol cm^−2^ at the end of October and gradually decreased to 1.8 × 10^18^ mol cm^−2^ towards the end of the simulation period.

MOPITT and IASI CO columns are not directly comparable, because different AK and *a priori* profiles are used in the retrieval. Applying a different AK to the model column explains the 40% lower peak MOPITT columns (figure 2*a*) compared with IASI columns (figure 2*b*). To illustrate the larger dependence of the MOPITT product on the prior, the prior CO total columns before and after applying the AK are shown in figure 2*c*. The IASI total columns before and after applying the AK are of similar magnitude, showing that the reported IASI observations are representative of the CO total column during this biomass burning event. However, the weekly MOPITT columns in October 2015, the month with the largest CO columns, are reduced from 3–3.5 × 10^18^ mol cm^−2^ to 2.2–2.5 × 10^18^ mol cm^−2^ when the AK is applied to the modelled columns. This points to a significant weight of the prior MOPITT climatological total column, which has a rather stable value of about 1.7 × 10^18^ mol cm^−2^ (pink line in figure 2*c*). This result also confirms the findings of George *et al.* [17], who showed that the IASI retrieval assigns a larger error to the prior CO profile, while MOPITT assigns a smaller error to a more realistic prior CO representation from a global climatology. They also suggested that IASI performs better than MOPITT at detecting unexpected extreme events, while MOPITT was found to have a better performance in detecting anthropogenic emissions due to the larger sensitivity near the surface.

**Figure 3.**
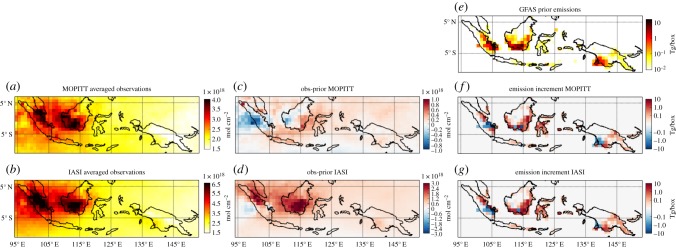
(*a*) MOPITT and (*b*) IASI observations averaged over each grid-cell and over the simulation period and (*c*,*d*) differences with respect to the simulated prior columns. (*e*) Spatial distribution of the CO emissions from fires over Indonesia and Papua, and increments when optimizing with (*f*) MOPITT and (*g*) IASI data.

MOPITT and IASI observations over Indonesia and Papua averaged over the simulation period (1 August–15 December) and over each grid-cell are shown in figure 3*a*,*b*. The largest mean columns are observed over the East of Sumatra and the South and West of Kalimantan, the largest island in Central Indonesia (figure 1). The CO columns reach 4 × 10^18^ mol cm^−2^ for MOPITT and 6.5 × 10^18^ mol cm^−2^ for IASI. This is a region of significant fire emissions, but also a region to which the westerly wind transports pollution from other parts of Indonesia and Papua. The model simulation with prior GFAS emissions overestimates the MOPITT columns over Sumatra and West Kalimantan, and underestimates in South and East Kalimantan, as well as in Eastern Indonesia and Papua (figure 3*c*). In the case of IASI (figure 3*d*), observations are mostly underestimated when using the prior emission, by up to 3 × 10^18^ mol cm^−2^ over South Kalimantan, and slightly overestimated west of Sumatra island.

Optimizing emissions by assimilating satellite observations leads to significant improvements between model and satellite observations, as shown in figure 2*a*,*b*. Posterior weekly averaged columns over Indonesia and Papua compare much better with satellite data than the model runs using prior emissions, but are still slightly underestimated for both MOPITT and IASI inversions.

Despite the differences in instrument, sampling, AK and observed columns, similar emission increments are found with respect to the prior (shown in figure 3*e*) when assimilating either IASI or MOPITT observations (figure 3*f*,*g*). Optimized emissions are decreased compared with GFAS in central Sumatra and part of Papua, and increased over most of Central Indonesia and West Papua island.

### (b) Emission estimates—region partitioning and time evolution

**Figure 4.**
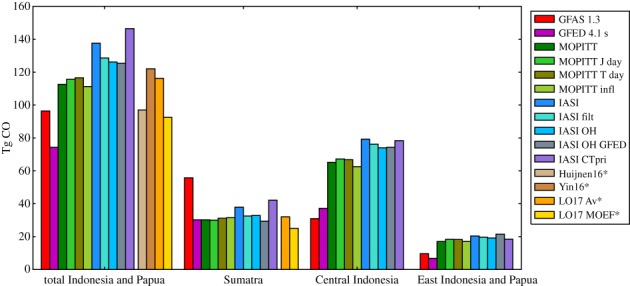
Total prior and posterior CO emissions by region during 15 August–15 November 2015. Also included are estimates from Akagi *et al.* [14] and Huijnen *et al.* [15] and scaled CO_2_ estimates from Heymann *et al.* [18] (LO17 Av and LO17 Indonesian Ministry of Environment and Forestry (MoEF)).

We report optimized emissions over Indonesia and Papua, summed over the period mid-August to mid-November, when most fires of 2015 took place. We neglect the first two weeks of simulation to avoid the influence of initial conditions and the final month, which is less constrained by observations. Total best estimates are 113 Tg according to our standard ‘MOPITT’ inversion and 138 Tg according to the ‘IASI’ inversion. Estimated emissions for entire Indonesia and Papua as well as the split over three regions according to figure 1 are presented in figure 4. Emissions are robust to choices concerning MOPITT observations and prior emission assumptions (‘MOPITT J day’, ‘MOPITT T day’, ‘MOPITT infl’, ‘IASI OH GFED’ and ‘IASI CTpri’; see §2 and electronic supplementary material for details). Somewhat larger differences are found when accounting for the effect of CO on the regional OH abundance and for the different daily coverage of the IASI and MOPITT data (‘IASI OH’ and ‘IASI filt’). Total emissions are about 10 Tg lower for these simulations than the estimate with default OH and using the full IASI dataset.

Looking at the sub-regions of Indonesia and Papua, the emissions for Sumatra are estimated in the range of 29–38 Tg, in agreement with GFED4s estimates but significantly lower than GFAS, which estimates total Sumatra emissions of 55 Tg CO in this period. For the Central Indonesian region, which mainly includes Kalimantan and Sulawesi islands, the emissions increase from the prior estimates of 37 Tg (GFED4s) and 30 Tg (GFAS1.3) to 63–67 Tg for the range of simulations using MOPITT and to 74–79 Tg for the simulations using IASI data. The emissions in East Indonesia and Papua also increase from the prior estimates of 7 Tg (GFED4s) and 10 Tg (GFAS1.3) to 17–21 Tg.

By adding the relative differences arising in the sensitivity simulations with respect to model parameters, we estimate roughly 10% uncertainty in our emission estimates from Indonesia and Papua based on either MOPITT or IASI. The main component is the uncertainty with respect to OH levels. This uncertainty can reach 30% for sub-regional estimates, where prior uncertainty and assumed observation error also become important. The sensitivity simulations cannot fully explain the difference between IASI and MOPITT results, pointing to a 10–20% bias between the two instruments during this event.

The range of total emissions we find for the Indonesian fires (113–138 Tg) is higher than prior estimates from emission models (74 Tg for GFED4s and 96 Tg for GFAS1.3). Our MOPITT-based estimate (113 Tg) is in very good agreement with the estimate from Yin *et al.* [16] (112 Tg CO), but is significantly higher than the estimate of Huijnen *et al.* [15] (96 Tg CO), though within their quoted uncertainty range of 18 Tg. These estimates were found by extrapolating from an estimated 84 Tg CO emission during September and October in the case of [15] to our analysis period, and by subtracting 10 Tg emissions from outside our analysis period from the yearly estimate of 122 Tg CO in the case of Yin *et al.* [16]. Both studies used MOPITT data to constrain CO emissions from the Indonesian fires. The discrepancy with Huijnen *et al.* [15] is likely due to the different OH fields used in their model and to their different optimization techniques. The OH fields in [15] are based on online chemistry calculations and take into account the OH perturbation caused by the fires (see electronic supplementary material). By contrast, Yin *et al.* [16] use the same climatological OH fields as our simulations, which are consistent with methyl chloroform observations [36,37]. We also tested the potential effect of modified OH concentrations due to fires on CO inversion results and found a 11 Tg lower CO emission related to a lowering of OH by peat fires (see electronic supplementary material).

Our CO emission estimates for Sumatra and total Indonesia also match those based on the [19] CO_2_ emissions. They estimated CO_2_ emissions using BA from Sentinel-1A synthetic aperture radar data and two different peat-maps (Avitable—Av and Indonesian Ministry of Environment and Forestry (MoEF)). To convert to CO emissions, we used GFED emission factors for forest fires and peat emission factors from Stockwell *et al.* [38]. Note that Papua New Guinea and small Indonesian islands were not included in their analysis; therefore, we do not show their results for the other two regions.

**Figure 5.**
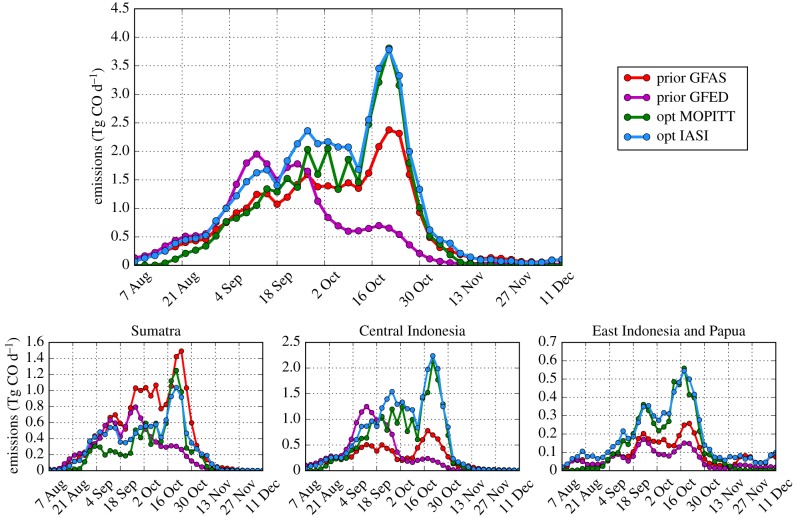
Prior and posterior emission evolution in time during the 2015 fires over Indonesia and Papua optimized using IASI and MOPITT satellite data.

Figure 5 shows the emission evolution in time over Indonesia and Papua, and their sub-regions. Most emissions are found in October in all regions, with particularly large emissions in the second half of October and a smaller emission peak occurring at the end of September and beginning of October. The results obtained with the IASI and MOPITT datasets are similar in terms of both time evolution and the split between regions. These features are robust with respect to the satellite dataset used and model settings (see electronic supplementary material).

## 4. Discussion

We have shown that robust CO emission estimates from large fire events can be derived from satellite data based on satellite data from IASI or MOPITT. We find about 20% difference in estimated emissions between inversions using MOPITT and IASI, respectively. Sensitivity tests performed show that some of the discrepancy might be related to the different data coverage between the two instruments. Other sensitivity tests showed that results are generally robust with respect to model set-up. However, a main source of uncertainty, about 8%, is related to the assumed OH sink in the model. We have shown here that OH concentrations are also sensitive to fire emissions, and taking this sensitivity into account may improve estimates of CO fire emissions in future inversions.

The amount of CO emission from fires depends on the amount of burned carbon and combustion efficiency. As peat fires emit two to four times more CO than forest fires per kg of fuel burned [14,15,39], the combustion efficiency is strongly dependent on the partitioning of burned carbon between peat and non-peat biomes. It is thus important to have a good estimate of peat area, particularly in Indonesia, where 60 Pg C are stored in the form of peat. Widespread peat fires, which develop in Indonesia during drought conditions associated with El Niño events, have a significant contribution to the exchange between terrestrial carbon stocks and the atmosphere, and partly offset the uptake of atmospheric CO_2_ by the biosphere.

Our estimates of CO emissions can be used to quantify the release of gaseous total carbon emissions to the atmosphere (which includes CO_2_, CO, CH_4_ and NMVOC). For this conversion, we need to use biomass burning emission factors, which are quite uncertain. Based on our range of results and a range of emission factors available in the literature [14,15,39], we find that a range of 0.35–0.60 Pg C was emitted from the 2015 fires in Indonesia and Papua.

Large discrepancies were found in our study between emission inventories and top-down CO emission estimates for the different sub-regions of Indonesia. Such discrepancies are often associated with uncertainties in burned carbon, which in the case of peat may also originate from uncertain peat depth. However, they can also be an indicator of innacurate biome attribution of fire emissions and highlight the need for further investigation of peat distribution. Both peat area and depth were found to have significant uncertainties in Indonesia [40]. Another indication of peat distribution uncertainties is the fact that Lohberger *et al.* [19] find about half of carbon emitted in West Papua during the 2015 fires to originate from peat, while GFED did not attribute any fires to peat burning in Papua during this event. Efforts to produce an accurate peat map for Indonesia are underway (http://indonesianpeatprize.com/).

The spatial scale to which results are analysed can potentially be improved beyond the regional size of the order of 1000 × 1000 km^2^ for which we have shown results here. The sensitivity of our model to regional emissions from Kalimantan, Sulawesi and Maluku (electronic supplementary material, figure S8) shows that the model is able to separate emission regions that are only 300 km away. With the Sentinel 5P TROPOMI satellite data becoming available [41], CO emissions can likely be constrained on even finer spatial and temporal scales. Continuous validation of satellite data remains important towards this end, and first tests of the TROPOMI CO data showed promising results in terms of data quality.

Looking at the temporal scale, the emission time evolution captured by our inversions is similar to the one of the GFAS inventory, although with different emission levels. GFED estimates a double-peaked emission evolution as well, but with much higher emissions at the end of September than during October. The reason for this difference is likely related to the different types of data used by the two inventories. GFAS uses FRP satellite data to estimate emissions. When these data are not available over a certain grid-cell because of cloud cover, GFAS assumes that fire emissions continue until a new measurement becomes available. The evolution of FRP over Indonesia and Papua is consistent with our posterior emissions (electronic supplementary material, figure S7), and this is likely the reason that GFAS finds a similar timing of peak emissions. GFED estimates monthly emissions from satellite measurements of BA and distributes emissions within the month according to FRP. Particularly in peat-dominated fires, BA might be more sensitive to the initial stages of burning and less sensitive to the continued burning that often occurs in the peat substrate. Therefore, the use of BA observations in areas where peat fires are important may not capture the actual timing of emissions. While the underlying reasons may be different, van der Laan-Luijkx *et al.* [6] showed that atmospheric information also indicated a later peak than GFED in the 2010 Amazon fire season.

Past forest loss in Sumatra and Kalimantan was found to be linked to deforestation and population growth [42]. Forest loss rates in Sumatra were significant since the 1960s, while in Kalimantan the rates started to grow only in the 1980s. In the economic development plan envisioned by the Indonesian government in 2014, West Papua was targeted as an area where significant agricultural development can be made in the near future [43]. We found double the CO emissions in East Indonesia and Papua than emissions reported by inventories. Forest loss in this region was about 6 times larger in 2015 than the 2001–2014 average, while in Sumatra and Kalimantan forest loss was comparable to the average of the previous 15 years (from www.globalforestwatch.org). Together with an increase in fire susceptibility related to climate change [44], the future agricultural development of West Papua might pose a significant fire risk. Satellite data of CO can be used towards monitoring the effectiveness of measures taken in reducing fire occurrence and spread.

CO satellite data products from IASI and MOPITT can complement existing fire indicators such as FRP, FCs and BA as part of operational systems such as CAMS (https://atmosphere.copernicus.eu/). Leip *et al.* [45] recommend monitoring greenhouse gas emissions by a combination of top-down and bottom-up methods. Similarly, we recommend combining current fire emission inventory techniques with top-down estimates based on atmospheric constraints. Integrating multiple data streams would improve emission estimates, fire monitoring and our general understanding on fire processes.

## Supplementary Material

Suplementary Material

## Supplementary Material

Evolution of IASI CO columns

## Supplementary Material

Evolution of MOPITT CO columns

## Data accessibility

Model output is available at https://trng-b2share.eudat.eu/records/01649b2e67b242d3a69d356f9b976789, and model code is available upon request from Narcisa Nechita-Banda (n.l.banda@uu.nl). Emission data from GFASv.1.3 were provided by the GFAS-CLIM project (BMWi FKZ 50EE1543). MOPITT Version 7 Level 2 data used for this research are available at the NASA Atmospheric Sciences Data Center (ASDC) datapool at https://eosweb.larc.nasa.gov/HPDOCS/datapool/. IASI is a joint mission of EUMETSAT and the Centre National d’Etudes Spatiales (CNES, France). The IASI CO product described in this paper is operationally distributed by Eumetcast, under the auspices of the Eumetsat Atmospheric Monitoring Satellite Application Facility (AC-SAF; http://ac-saf.eumetsat.int); scientific data and quick-looks are available from the Aeris data infrastructure (http://iasi.aeris-data.fr).
