# Supplementary material for: Monitoring emissions from the 2015 Indonesian fires using CO satellite data
Source: Philos Trans R Soc Lond B Biol Sci. 2018 Oct 8;373(1760):20170307. doi: 10.1098/rstb.2017.0307 (PMC6178426; doi:10.1098/rstb.2017.0307)
Supplement: Suplementary Material [file rstb20170307supp1.docx]

**Supplementary material**

**Cross-validation of MOPITT and IASI results**

Cross-validation of the ‘IASI’ inversion using MOPITT data, and of the ‘MOPITT’ inversion using IASI (Figures S1) show that ‘MOPITT’ inversion underestimates significantly the IASI data, while the ‘IASI’ inversion overestimates the MOPITT data. This is to be expected from the fact that MOPITT-optimized emissions are lower than IASI-optimized emissions, and points to a potential bias between the two instruments during this event.

**Sensitivity to satellite data products & filtering**

One of the differences between MOPITT and IASI is that MOPITT also measures in the near infra-red (NIR) in addition to the thermal infrared (TIR) at which IASI measures as well. A second difference is that for IASI we use day-time data, while for MOPITT both day-time and night-time data are used in our inversion. To test the potential implications of these choices, we made sensitivity tests using the NIR-TIR day-time only data (‘MOPITT J day’), and TIR day-time only data (‘MOPITT T day’), in additional to the inversion using both day-time and night-time NIR-TIR data (‘MOPITT’). Results obtained with the three datasets are very consistent, both in terms of total emissions, which differ only by a few Tg, as well as in terms of location and temporal evolution (Figure S2). We can therefore conclude that the day-time and night-time data are consistent, as well as emissions inferred from the TIR and NIR-TIR products. A remaining source of uncertainty is the different sampling time of the two instruments. A diurnal temporal profile of the emissions is not implemented in the model, and this might cause some uncertainty in the resulting emissions. However, the fact that the MOPITT results using both day-time and night-time data are consistent with those using day-time data alone indicate that a diurnal profile will likely not affect the results significantly.

Another choice that might affect our results is the satellite data standard deviation assumed in the model to account for error correlation due to large density of satellite measurements. An error inflation factor of $\sqrt{50}$ is applied for IASI, but not for MOPITT. Applying an error inflation factor of $\sqrt{50}$ to the MOPITT observations in the ‘MOPITT infl’ inversion did not affect significantly the results (Figures 4 and S3).

Remaining differences might be due to differences between the IASI and MOPITT data, such as instrument design, vertical sensitivity, and data coverage. We further tested the hypothesis that the different data coverage due to the much narrower swath and stricter cloud filtering of MOPITT compared to IASI might influence the results. Since biomass burning emits significant aerosol amounts together with CO, data in thicker areas of the plume might be filtered out by the filtering procedure of MOPITT. We filtered out the IASI observations from locations that are farther than 1° in terms of longitude + latitude from MOPITT observations on the same day. We note here that IASI and MOPITT have a one hour difference in sampling time, which can still lead to some differences in the sampling of the plume between the MOPITT and the filtered IASI dataset. The resulting emissions from this filtered IASI dataset are somewhat lower than the full IASI simulation, 129 Tg in total, with most of the difference originating in Sumatra. For the other regions, the temporal evolution and total emissions remain close to the original IASI result. In Sumatra, the emission evolution is in between that of IASI and that of MOPITT (Figure S4), showing that the results might indeed be slightly influenced by data coverage. This is also the region where both IASI and MOPITT have the lowest number of observations per grid cell on average, possibly because more data were filtered out due to aerosol contamination.

**Sensitivity to modelling setup**

CO is mainly removed by its reaction with OH, having a lifetime of about 1-2 months in the atmosphere. In addition to CO, fires also emit NO_X_, CH_4_ and NMVOCs, which influence OH recycling and consumption. (1) have shown that OH concentrations are inversely correlated with the CO/NO_X_ ratio. Therefore, a peat fire has the potential to decrease OH concentrations significantly, due to a high CO/NO_X_ emission ratio related to incomplete burning.

The optimised emissions would be sensitive to the CO sink assumed in the model, which typically uses climatological OH fields from (2). In the ‘IASI OH’ inversion we evaluate the potential magnitude of the effect of OH on posterior emissions by using adjusted OH fields. The OH climatological fields were scaled with the daily OH ratio between two chemistry simulations with and without the emissions from peat fires performed with C-IFS (3). In the chemistry simulations, we adjusted the GFED emissions using the (4)) emission factors for peat, which have a higher CO/NO_X_ ratio that typically used in GFED.

The resulting OH anomalies are largest in October and above Sumatra island, with local monthly anomalies of more than 30% (Figure S6). The resulting total emissions are about 11 Tg lower than the results from the ‘IASI’ simulation using climatological OH, with the largest adjustments over Sumatra (Figures 4 and S7). This sensitivity can be seen as a maximum effect that changes in OH due to fires can have on posterior emissions. Forest fires, which typically have a lower CO/NO_X_ ratio, occurring in Indonesia at the same time with peat fires, increase OH and counteract the OH decrease due to peat fires (results not shown).

The posterior emissions might also be sensitive to the prior emissions used in the inversion system. We therefore performed two extra simulations, ‘IASI OH GFED’ using GFED as prior biomass burning inventory, and ‘IASI CTpri’ using constant prior emission evenly distributed over the land regions. The resulting posterior emission evolution remains largely unaffected by the choice of the prior, while regional emission totals differ by 2-3 Tg during 15 August to 15 November 2015. Although the two emission inventories have a significantly different evolution in time, the evolution of the posterior emissions is similar, with peak emissions in the second half of October (Figure S8).

1. Dalsøren SB, Isaksen IS a. CTM study of changes in tropospheric hydroxyl distribution 1990–2001 and its impact on methane. Geophys Res Lett. 2006 Dec 13;33:L23811.

2. Spivakovsky CM, Logan JA, Montzka SA, Balkanski YJ, Foreman-Fowler M, Jones DBA, et al. Three-dimensional climatological distribution of tropospheric OH: Update and evaluation. J Geophys Res Atmos [Internet]. 2000 Apr 16 [cited 2017 Oct 4];105(D7):8931–80. Available from: http://doi.wiley.com/10.1029/1999JD901006

3. Flemming J, Huijnen V, Arteta J, Bechtold P, Beljaars A, Blechschmidt A-M, et al. Tropospheric chemistry in the Integrated Forecasting System of ECMWF. Geosci Model Dev [Internet]. 2015 Apr 7 [cited 2018 Feb 14];8(4):975–1003. Available from: http://www.geosci-model-dev.net/8/975/2015/

4. Stockwell CE, Jayarathne T, Cochrane MA, Ryan KC, Putra EI, Saharjo BH, et al. Field measurements of trace gases and aerosols emitted by peat fires in Central Kalimantan, Indonesia, during the 2015 El Ni??o. Atmos Chem Phys. 2016;

Table S1 Simulation setup

| Simulation name | Satellite data | Prior | OH | Error inflation |
| --- | --- | --- | --- | --- |
| MOPITT | MOPITT NIR-TIR day- and night-time | GFAS 1.3 |  |  |
| IASI | IASI day-time | GFAS 1.3 |  | $\sqrt{50}$ |
| MOPITT J day | MOPITT NIR-TIR day-time | GFAS 1.3 |  |  |
| MOPITT T day | MOPITT TIR day-time | GFAS 1.3 |  |  |
| MOPITT infl | MOPITT NIR-TIR day- and night-time | GFAS 1.3 |  | $\sqrt{50}$ |
| IASI filt | IASI day-time filtered to match daily MOPITT spatial coverage | GFAS 1.3 |  | $\sqrt{50}$ |
| IASI OH | IASI day-time | GFAS 1.3 | Scaled based on C-IFS | $\sqrt{50}$ |
| IASI OH GFED | IASI day-time | GFED 4.1s | Scaled based on C-IFS | $\sqrt{50}$ |

**
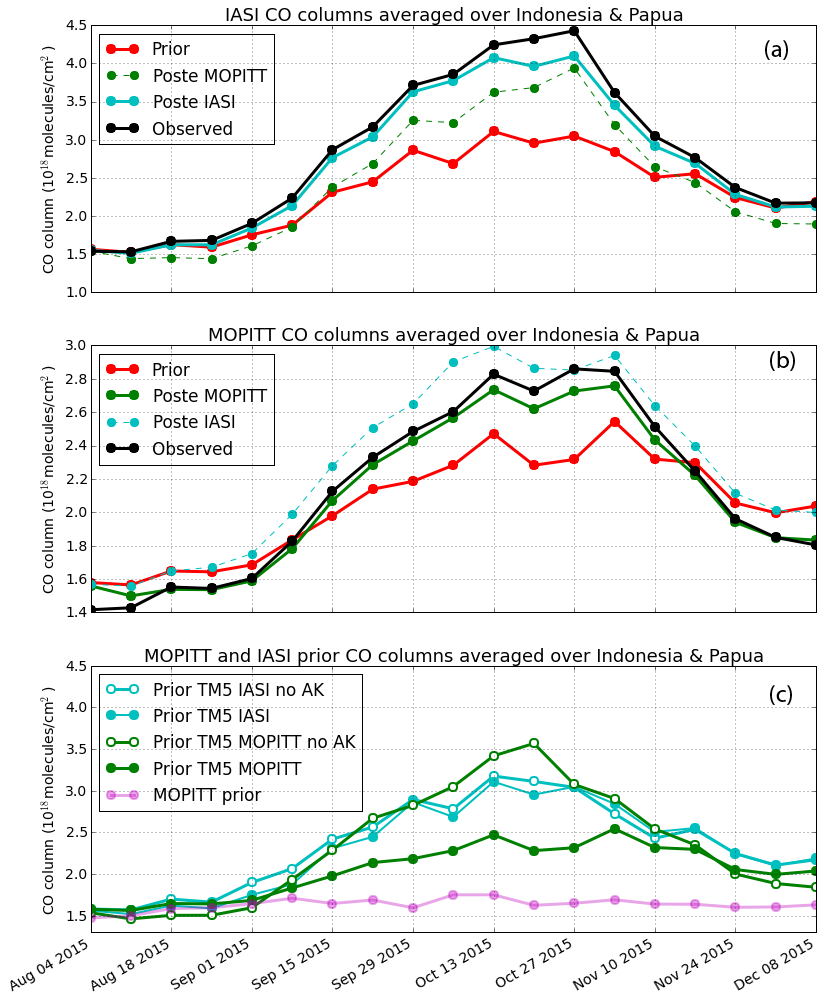
**

Figure S1 Same as Figure 2a and 2b, including cross-validation of MOPITT and IASI. The IASI columns simulated by the MOPITT inversion are shown in a) (dotted green line) and the MOPITT columns simulated by the IASI inversion in b) (dotted blue line).


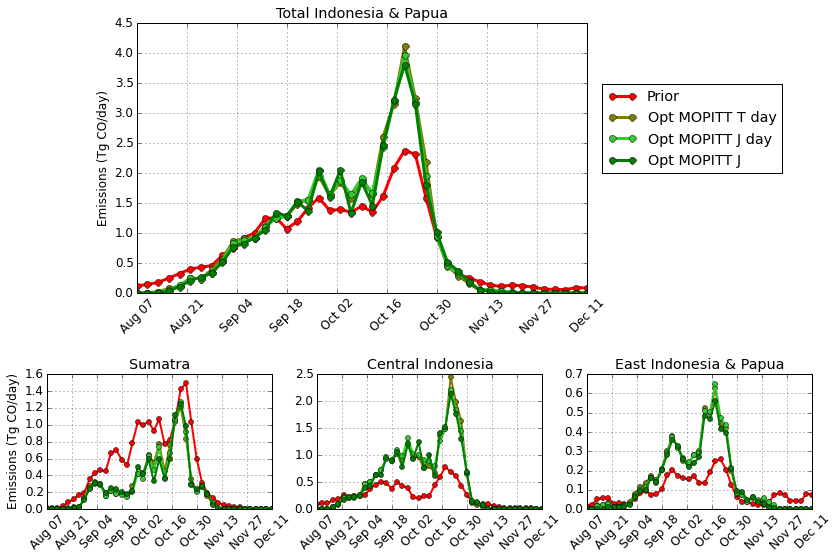


Figure S2. As in Figure 5, for different MOPITT products: TIR only product day-time data (MOPITT T day), joint NIR-TIR product day-time data (MOPITT J day), and joint NIR-TIR both day-time and night-time data (MOPITT J). MOPITT J is the MOPITT product discussed in the main paper.


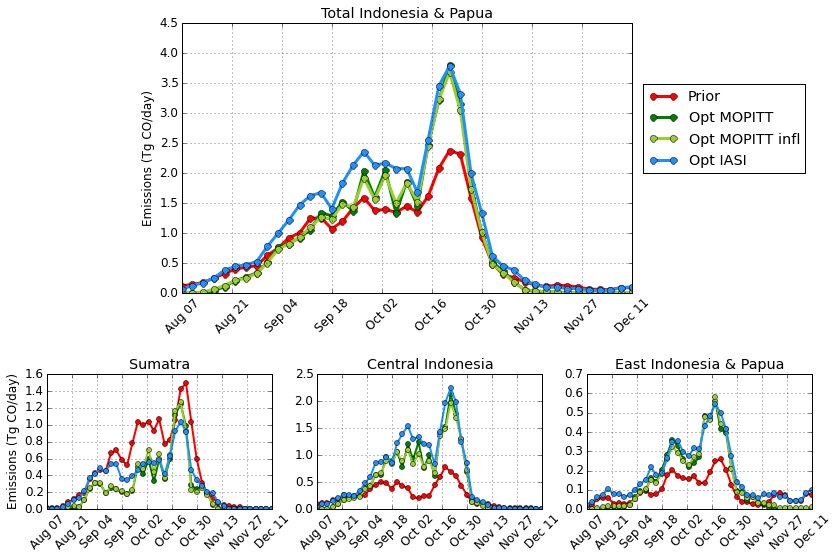


Figure S3 As in Figure 5, also including the results from the ‘MOPITT infl’ simulation, where the MOPITT observation variance is enhanced 50 times.


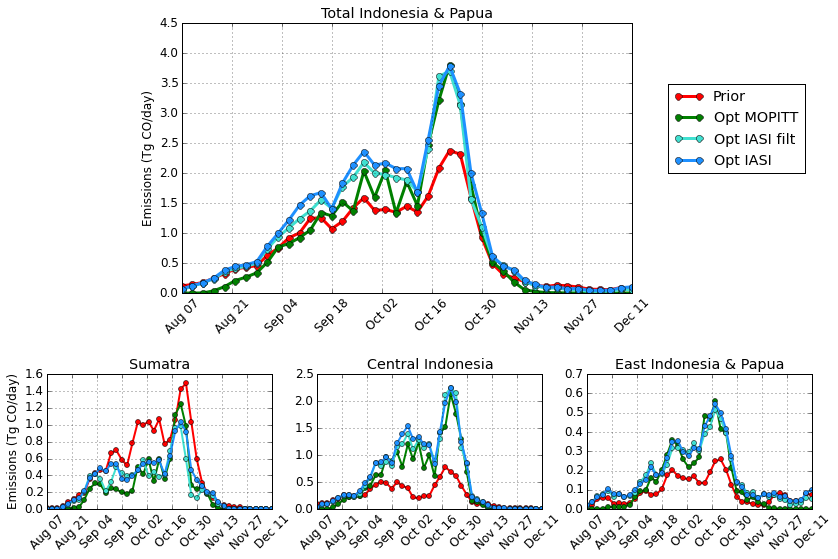


Figure S4. As in Figure 5, with the addition of posterior emissions optimized using filtered IASI data. IASI data from locations with no MOPITT measurement within 1° latitude + longitude on the same day was filtered out.


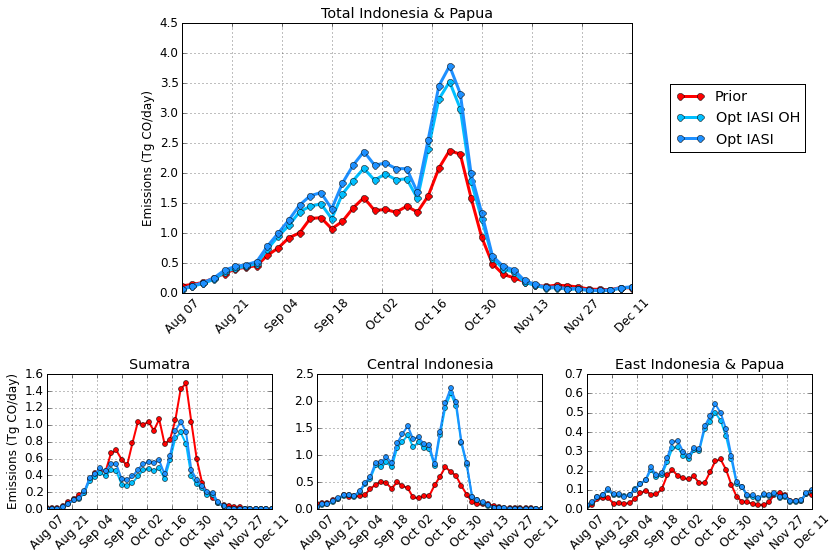


Figure S5. As in Figure 5, showing optimized emissions based on IASI data with two different OH fields. ‘IASI’, also shown in the main paper, uses the default climatological OH fields in TM5. In ‘IASI OH’ we scaled the standard OH fields to account for OH changes due to the fire activity.


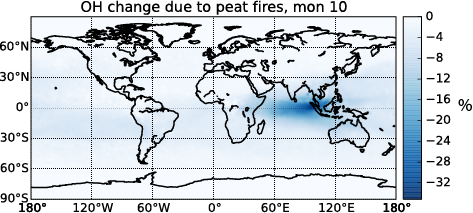

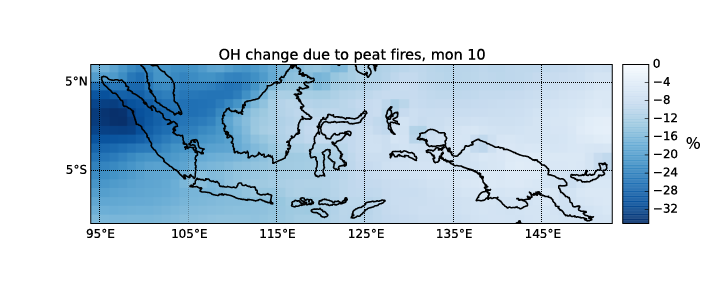


Figure S6. Relative changes in OH in October 2015 due to the peat fires in Indonesia and Papua, simulated with C-IFS.


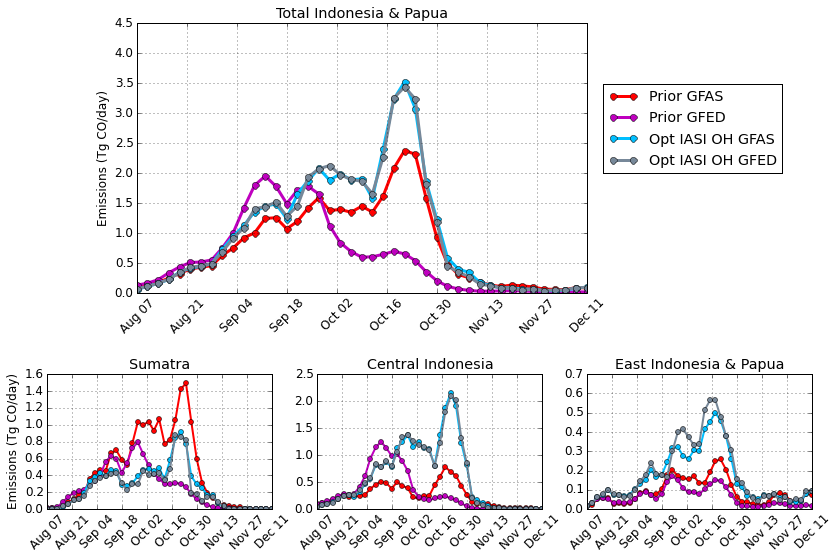


Figure S7. As in Figure S5, prior and posterior CO emissions using modified OH fields, and two different priors - GFAS 1.3 and GFED 4.1s.


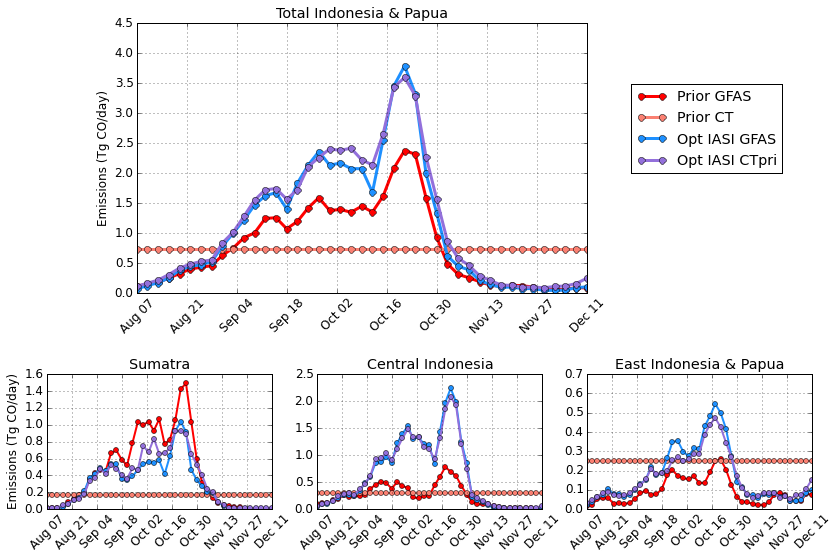


Figure S8. As in Figure 5, prior and posterior CO emissions using IASI starting from GFAS 1.3 and constant prior emissions.


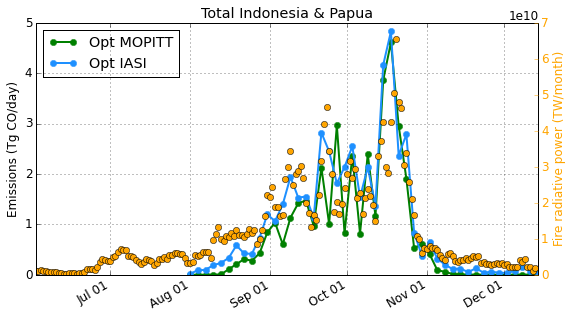


Figure S9 Time evolution of optimized emissions and observed fire radiative power (orange) over Indonesia and Papua during June to December 2015.


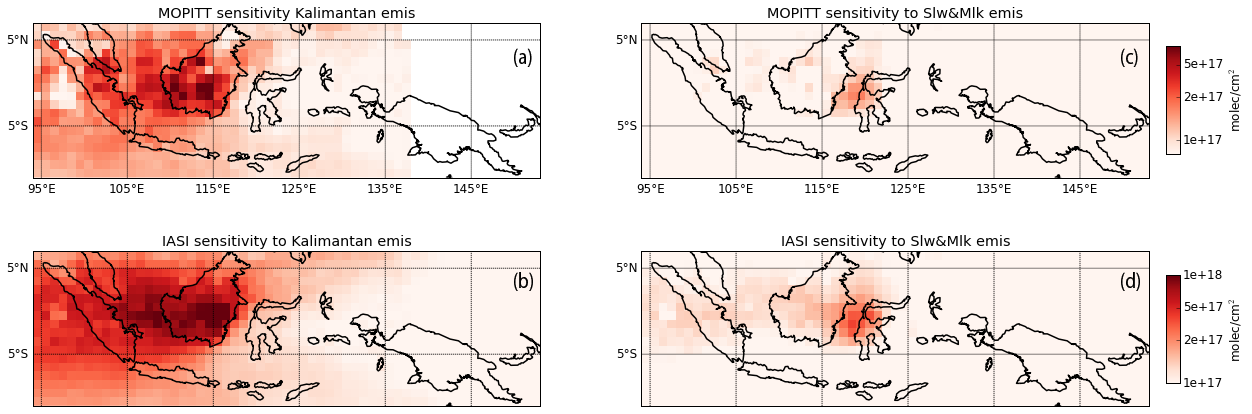


Figure S10 Differences in MOPITT and IASI CO total columns over Indonesia and Papua, averaged over 1 August to 15 December 2015, due to emission perturbations over Kalimantan (a and b) and over Sulawesi and Maluku islands (c and d). The emission perturbations applied are equal to the emission differences between posterior ‘IASI’ and prior emissions.
