# Supplementary figures and images for: Monitoring emissions from the 2015 Indonesian fires using CO satellite data
Source: Philos Trans R Soc Lond B Biol Sci. 2018 Oct 8;373(1760):20170307. doi: 10.1098/rstb.2017.0307 (PMC6178426; doi:10.1098/rstb.2017.0307)

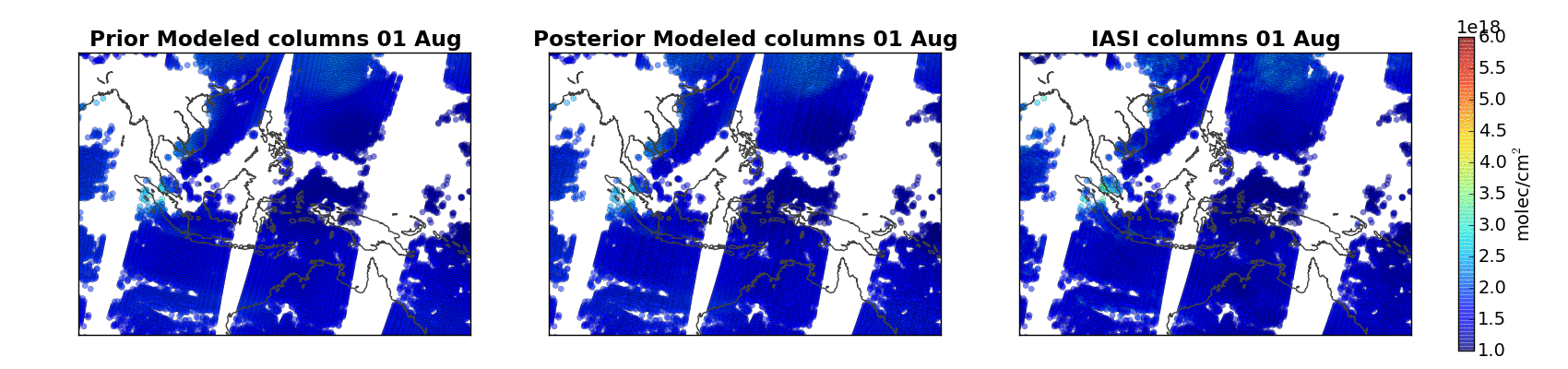

Supplement: Evolution of IASI CO columns [file rstb20170307supp2.gif]

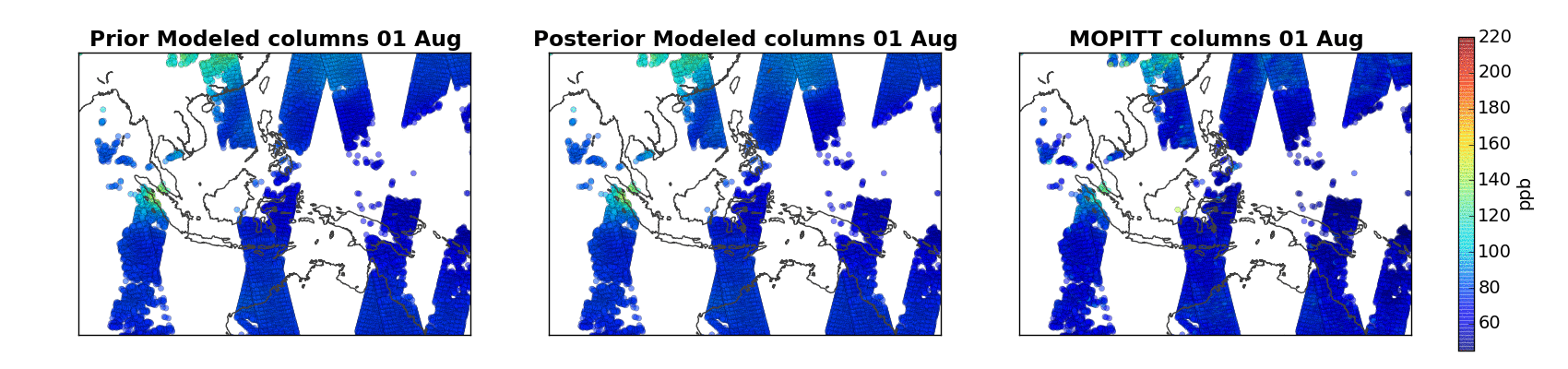

Supplement: Evolution of MOPITT CO columns [file rstb20170307supp3.gif]
